# Assessment of left atrial structural remodeling in patients with cryptogenic stroke - lessons learned from LGE-MRI

**DOI:** 10.1186/1532-429X-18-S1-P202

**Published:** 2016-01-27

**Authors:** Christian Mahnkopf, Marcel Mitlacher, Johannes Brachmann

**Affiliations:** grid.419808.c0000000403907783Klinikum Coburg, Coburg, Germany

## Background

Cryptogenic embolic strokes of undetermined source (ESUS) are thought to comprise about 25% of all ischemic strokes. Late-Gadolinium MRI (LGE-MRI) allows detection and quantification of left atrial structural remodeling (LA-SRM). We sought to compare the degree of LA-SRM using LGE-MRI in patients with ESUS and in patients with embolic stroke of know origin, especially in those with atrial fibrillation (AFIB).

## Methods

**Figure 1:**
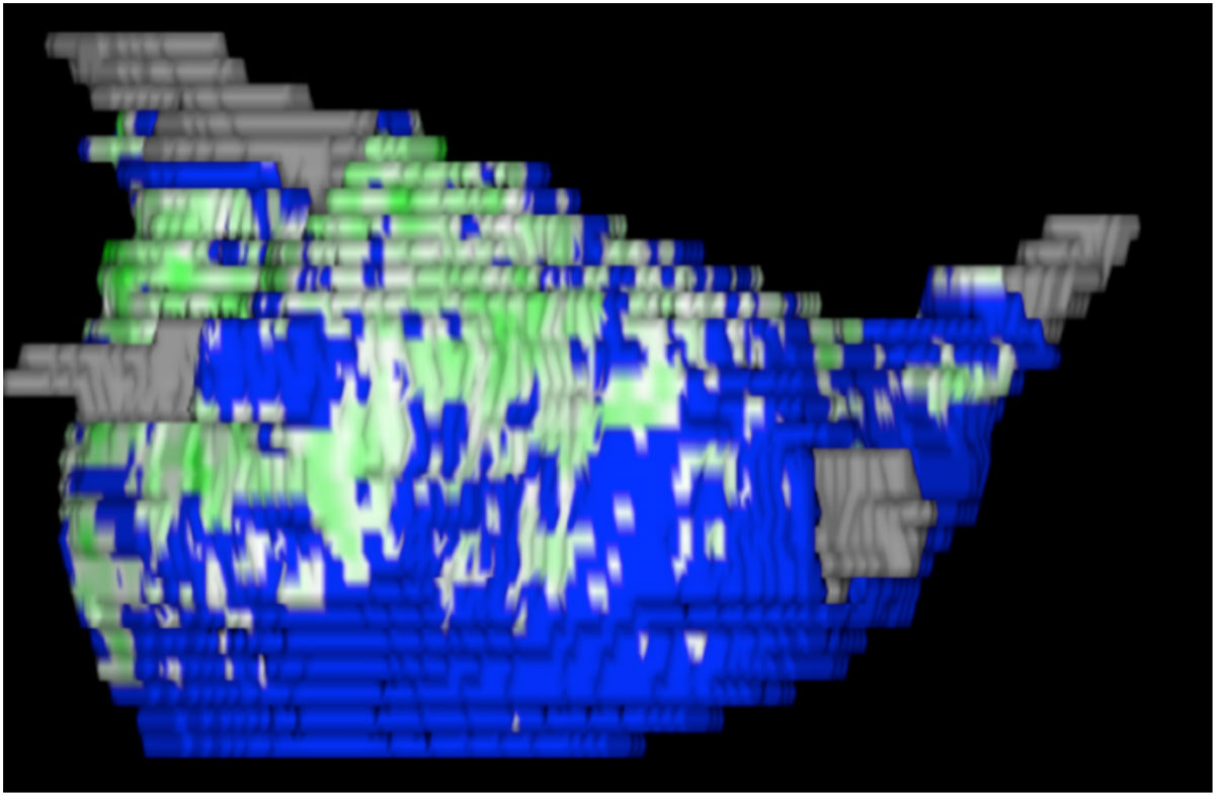
**3D-Model of the left atrium**. Example of a patient with extensive LA-SRM. Blue reflects healthy LA-tissue, while fibrotic areas are given in green. Pulmonary veins in great. PA-view (Merisight TM).

A total of fifty patients (31male (62%), Age 61.1 ± 14.2 years) with TIA or Stroke underwent LGE-MRI of the left atrium within 4 days after the event to assess for LA-SRM. Each LGE-MRI was segmented by isolating the LA wall and quantified for the relative extent of fibrotic remodeling using the Corview-Software (Merisight ™, Figure 1). Brain-CT or MRI, TEE, Sonography of the cerebral blood vessels and 24 hour ECG were performed in all patients.

## Results

**Figure 2:**
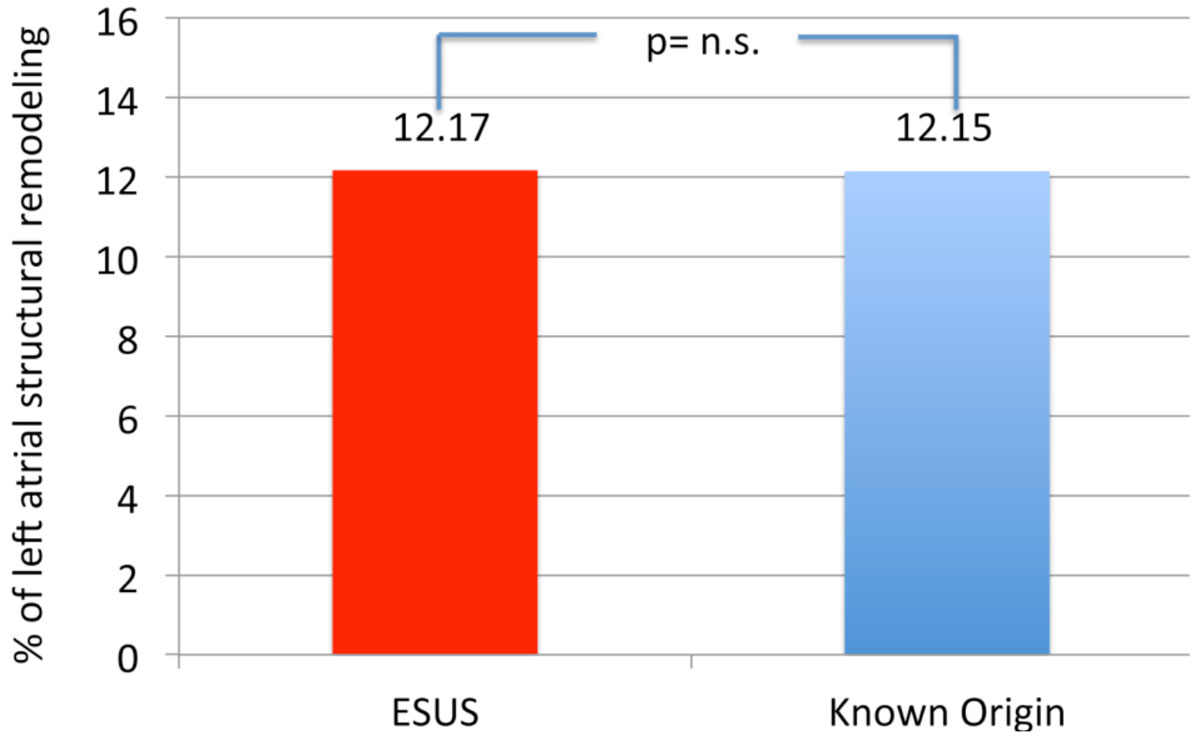
**Degree of LA-SRM in patients with embolic strokes of undetermined source (red column) and in patients with stroke of known origin (blue column)**.

**Figure 3:**
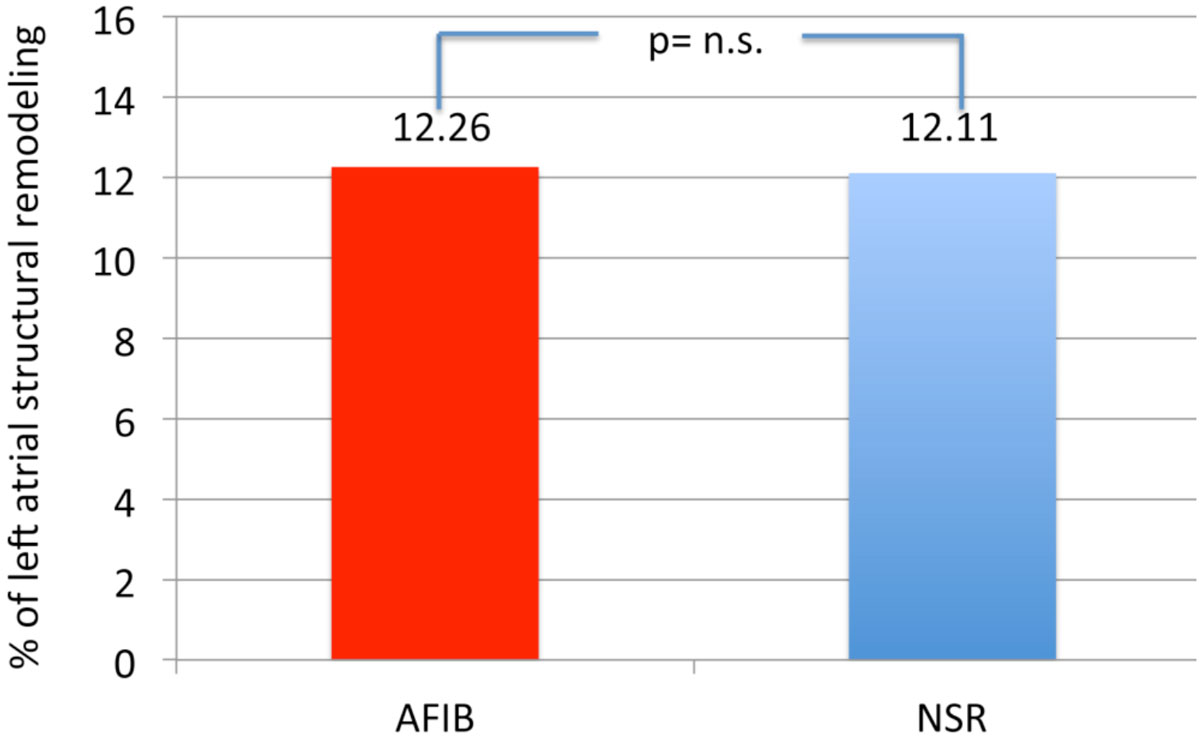
**Degree of LA-SRM in patients with embolic stroke and atrial fibrillation (red column) and in embolic stroke patients with normal sinus rhythm (blue column)**.

A total of 24 patients (48%) were specified with the diagnosis of ESUS, while a reason for the stroke event (AFIB, significant carotids stenosis, LAA-thrombus, persistent foramen ovale) was found in 26 patients (52%). The degree of left atrial remodeling was comparable in both groups (12.17 ± 5.23 vs. 12.15% ± 5.46%; p = 0.993; Figure 2). Overall, 15 patients (30%, 11 males) had a history of or were currently found with atrial fibrillation as a major reason for embolic stroke. Degree of LA-SRM (12.26% ± 6.4%) was comparable to those stroke patients with sinus rhythm (12.11 ± 4.85, p=n.s., Figure 3).

## Conclusions

From our preliminary results the degree of left atrial structural remodeling detected using LGE-MRI is comparable in patients with know origin of stroke and in those with so-called cryptogenic stroke. Thus, the extent of LA-SRM appears to play a critical role in the pathophysiology of embolic stroke and should be considered in the diagnosis, treatment, and risk stratification in stroke patients.

